# Lightweight 3D Lithiophilic Graphene Aerogel Current Collectors for Lithium Metal Anodes

**DOI:** 10.3390/ma17071693

**Published:** 2024-04-07

**Authors:** Caili Guo, Yongjie Ge, Piao Qing, Yunke Jin, Libao Chen, Lin Mei

**Affiliations:** 1 State Key Laboratory of Powder Metallurgy, Central South University, Changsha 410083, China; 15367485939@163.com (C.G.); 203301004@csu.edu.cn (P.Q.); jinyunke2022@163.com (Y.J.); 2 Key Laboratory of Carbon Materials of Zhejiang, College of Chemistry and Materials Engineering, Wenzhou University, Wenzhou 325035, China; geyongjie1220@wzu.edu.cn; 3 Foshan Lifriend New Energy Co. Ltd., Foshan 528244, China

**Keywords:** graphene, 3D current collectors, lightweight, lithium metal anodes

## Abstract

Constructing three-dimensional (3D) current collectors is an effective strategy to solve the hindrance of the development of lithium metal anodes (LMAs). However, the excessive mass of the metallic scaffold structure leads to a decrease in energy density. Herein, lithiophilic graphene aerogels comprising reduced graphene oxide aerogels and silver nanowires (rGO-AgNW) are synthesized through chemical reduction and freeze-drying techniques. The rGO aerogels with large specific surface areas effectively mitigate local current density and delay the formation of lithium dendrites, and the lithiophilic silver nanowires can provide sites for the uniform deposition of lithium. The rGO-AgNW/Li symmetric cell presents a stable cycle of about 2000 h at 1 mA cm^−2^. When coupled with the LiFePO_4_ cathode, the assembled full cells exhibit outstanding cycle stability and rate performance. Lightweight rGO-AgNW aerogels, as the host for lithium metal, can significantly improve the energy density of lithium metal anodes.

## 1. Introduction

Lithium metal is regarded as one of the most promising candidates for next-generation high-energy-density batteries owing to its high theoretical capacity (3860 mA h g^−1^) and low potential (−3.04 V vs. SHE), and thus has attracted considerable research attention in recent years [1,2,3,4]. However, the practical application of lithium metal anodes is still hindered due to the uncontrollable dendritic growth of lithium [5,6,7,8]. The accumulation of “dead” Li increases the risk of internal short circuits by piercing the separators during cyclic processes [9]. Furthermore, the deposition of lithium leads to electrode volume expansion, causing mechanical stress, electrode cracking and loss of electrical contact. Uneven lithium deposition leads to poor cycling stability and low Coulombic efficiency, further diminishing battery performance. In addition, the huge volume fluctuation and irregular deposition in lithium metal anodes result in the fracture of an unstable solid-electrolyte interface (SEI), which in turn causes the exposure of fresh lithium metal and increases electrolyte consumption [10]. Addressing these challenges is essential for the development of high-performance lithium metal batteries.

To solve the above problems, researchers have proposed multiple effective strategies, such as constructing artificial SEIs [11,12,13] and interface protection layers [14,15,16,17], optimization of electrolyte components (Li salts, organic solvents, and functional additives) [18,19,20,21], constructing three-dimensional (3D) frameworks [22,23,24,25], using solid-state electrolytes [26,27,28,29], and preparing composite Li metal anodes [30,31,32], etc. Among these, 3D skeletons with a high specific surface area play a key role in reducing local current density, retarding the initiation of lithium dendrite formation. Furthermore, the micro-porous structure of the 3D skeleton serves as holder for lithium deposition, effectively alleviating the volume change during the charge–discharge process [33,34,35].

However, the utilization of commercial metallic foam (e.g., copper, nickel) as 3D current collectors, with natural lithiophobic features, demonstrates uneven lithium deposition [36]. It has been suggested that introducing lithiophilic materials can improve the lithiophilicity of 3D current collectors, thus inducing uniform Li-ion flux and lithium deposition in the pore structure [37,38,39]. In recent years, significant advancements have been achieved in suppressing lithium dendrite formation and enhancing the stable cycling performance of lithium metal anodes. Qing et al. prepared a robust porous 3D nickel skeleton with lithiophilic Ni_3_N nanocoatings (Ni_3_N@NS) by the facile powder metallurgical and plasma-enhanced chemical vapor deposition method [40]. The symmetrical cell cycled steadily over 1000 h at 2 mA cm^−2^. However, the utilization of conventional metal materials with excessive mass poses a challenge to the advancement of high-energy-density batteries [41,42,43]. Carbon materials have attracted attention due to their lightweight properties. The CE of the half cell with carbon nanotubes acting as the 3D current collector remained at 90% over 150 cycles at 1 mA cm^−2^ [44]. The 3D-printed graphene oxide framework reduced the local current density and supplied space for the deposition of Li, enabling a long-term cycling stability of 1600 h at 1 mA cm^−2^ [45]. Despite the good modification effect, poor mechanical stability will accelerate the failure of the battery.

Because of its excellent properties, graphene has a good application prospect in electrochemical energy storage materials. However, graphene is difficult to produce in large quantities. It can be replaced with reduced graphene oxide (rGO), which has similar properties as graphene in many ways [46]. rGO can be obtained by chemical reduction and thermal reduction of graphene oxide. rGO prepared through the reduction process has many defects, including vacancies, edges, deformations, and residual oxygen-containing functional groups, which lead to low-to-medium quality [47]. But rGO is more than adequate when used as the host for a lithium metal anode. Like most carbon materials, rGO is lithiophobic. It has been reported that the nucleation overpotential of lithium on silver is close to zero [38], so we choose silver to enhance the material’s lithiophilicity.

In this study, we propose the utilization of a lithiophilic reduced graphene oxide aerogel material with silver nanowires as a host for lithium deposition. The material combines the advantages of the rGO aerogel and silver nanowires. The rGO-AgNW material exhibits lightweight characteristics, allowing for a reduction in the proportion of the current collector in the electrode, thereby effectively enhancing the energy density of the anode. The large specific surface area of the rGO aerogel can effectively reduce the local current density, and the internal micro-pores provide space for lithium deposition. The integration of lithiophilic silver facilitates more sites for lithium nucleation, which can achieve uniform lithium deposition and ensure the stable cycling of the anode electrode. In addition, silver nanowires can also enhance the structural stability of the rGO aerogel compared to nanoparticles. This novel approach of lightweight 3D lithiophilic current collectors could facilitate the application of high-energy-density batteries.

## 2. Materials and Methods

### 2.1. Fabrication of Reduced Graphene Oxide Aerogels with Silver Nanowires

The graphene oxide dispersion (GO, XFNANAO) in ultrapure water was firstly centrifuged at 3000 r/min and the upper liquid was centrifuged at 8000 r/min, both for 30 min. Then, the lower part was collected, ultrasonically mixed, and its concentration was calibrated. The dispersion was diluted to 2.5 mg/mL by adding ultrapure water. Sodium ascorbate (C_6_H_7_NaO_6_, Aladdin) was added at a mass ratio of 1:1 to GO. Silver nanowires, synthesized according to the previous report [48] and making up 10%, 20%, and 50% of the mass of GO, were added. The suspension after mixing was divided into reagent bottles with a diameter of 20 mm, 1 mL each. The reagent bottles were then transferred to the oven and maintained at 95 °C for 6 h, during which the GO was partially reduced. The obtained hydrogels were washed with a mixture of deionized water and ethanol (4:1 *v*/*v*) to remove residual chemicals. Finally, the obtained hydrogels were placed in the refrigerator to freeze and were then freeze-dried in a freeze dryer set at −40 °C and a vacuum of 20 Pa for 12 h to remove absorbed water and ethanol.

### 2.2. Materials Characterization

X-ray diffraction (Smartlab SE working at 40 kV and 40 mA, Cu K_α_ radiation, *λ* = 0.154 nm) was used to analyze the phase compositions with 2θ ranging from 10° to 90° at the scanning rate of 5° min^−1^. The surface components were analyzed by X-ray photoelectron spectroscopy, carried out on a Thermo Scientific K-Alpha working at 12 kV with the Al K_α_ radiation (*hv* = 1486.6 eV). Raman spectra were collected from 100 to 3500 cm^−1^ on a Raman spectrometer (Xplora, Horiba) at a laser wavelength of 532 nm and power of 5 mW. The thermo-gravimetric diagram of rGO-20AgNW was measured with a NETZSCH TG 209F1 Libra analyzer, and the test temperature range was 25–800 °C with a heating rate of 10 °C min^−1^ in an air atmosphere. We used a Micromeritics ASAP 2460 instrument to measure the N_2_ adsorption/desorption isotherms at 77.3 K and applied the Brunauer–Emmett–Teller (BET) method in calculating the specific surface area of samples. The microstructure images and elemental analyses of samples were conducted by field-emission scanning electron microscopy (SEM) (MIRA3 LMH, Chech, TESCAN) and attached energy-dispersive X-ray spectroscopy (EDS). The measurement of Young’s modulus was conducted on a tensile measurement machine (LD23.501, LSD), and the maximum compressive deformation of the samples was 80%.

### 2.3. Electrochemical Measurements

We assembled the CR2016 coin cells in an Argon-filled glovebox. The separator was Celgard 2400 membrane. An amount of 1 M lithium bis (trifluoromethanesulfonyl) imide (LiTFSI) in 1, 3-dioxolane (DOL) and dimethoxymethane (DME) in a volume ratio of 1:1 with 2% LiNO_3_ additives was the electrolyte. All cells were tested on the Neware BTS82 multichannel battery test system. The rGO aerogel with/without silver nanowires was directly used as a working electrode. For the measurement of CE, the aerogel was deposited for 6 h and charged to 0.5 V at 0.5 mA cm^−2^ to remove contaminants and form a stable SEI, then a fixed amount of Li (1 mAh cm^−2^) was deposited onto the aerogel and stripped away at up to 0.5 V at the current density of 2 mA cm^−2^. For the symmetric cell test, five pre-cycles were firstly employed between 0.01 V and 1.5 V and lithium was pre-plated onto the working electrode, both at a current density of 0.5 mA cm^−2^, and the cells were then cycled at different conditions (1 and 4 mA cm^−2^ for 1 mAh cm^−2^, 1 mA cm^−2^ for 5 mAh cm^−2^). The electrochemical impedance spectroscopy (EIS) of the symmetric cell was performed on a Princeton PARSTAT 4000 (AMETEK Co. Ltd., Shanghai, China) with a frequency from 100 kHz to 0.1 Hz and an amplitude of 5 mV.

LiFePO_4_ (LFP) powders, carbon black, and polyvinylidene fluoride (PVDF) with a mass ratio of 8:1:1 were mixed in a solvent of N-methyl-2-pyrrolidone (NMP). The slurry was pasted on aluminum foil, which was then placed in a vacuum oven and dried at 110 °C for 12 h. After that, the LFP-covered aluminum foil was punched into pieces of disks (*Φ* = 12 mm). The areal mass loading of LFP was approximately 1.4 mg cm^−2^. Composite electrodes pre-stored with 3 mAh cm^−2^ of Li were used as the anodes. The assembled full cells were first cycled five times at 0.1 C and were further cycled at 1 C (1 C =170 mA g^−1^), both between 2.4 and 4.0 V, to test the long cycle performance. The rate performance of full cells was tested at 0.5, 1, 2, 5, and 10 C.

## 3. Results and Discussion

**Figure 1:**
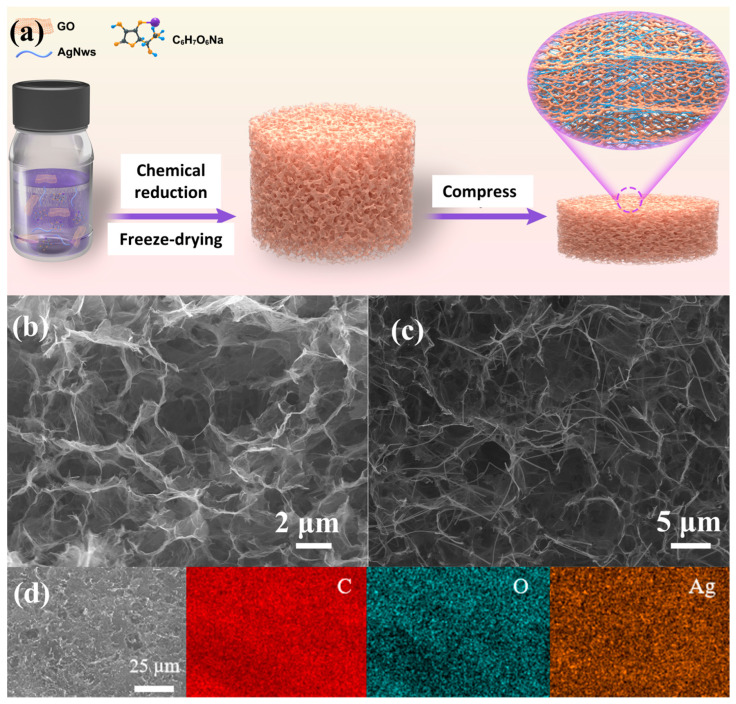
(**a**) Schematic of the preparation of reduced graphene oxide aerogel with silver nanowires. Cross-sectional SEM images of (**b**) rGO aerogel and (**c**) rGO-AgNW aerogel. (**d**) EDS element mapping of the rGO-AgNW aerogel.

The lightweight 3D lithiophilic graphene aerogel current collectors with AgNW are synthesized through the chemical reduction method, as shown in Figure 1a. The graphene oxide sheets transform from hydrophilic to hydrophobic when the oxygen-containing functional groups are reduced by sodium ascorbate, and self-assemble into a three-dimensional (3D) structure [49]. After chemical reduction and freeze-drying, the rGO-AgNW aerogel was compressed to a certain thickness and then directly acted as the lithium deposition host (Figure S1). The morphology of the aerogel was studied by SEM. As shown in Figure 1b, rGO is pleated, and there are relatively uniformly sized pores distributed inside the material, which can provide space for lithium deposition during circulation. The silver nanowires are evenly distributed around the edge of pores, fixed by graphene folds (Figure 1c). Elemental mapping of the rGO-AgNW aerogel reveals elemental Ag homogeneously distributed in the C matrix (Figure 1d), suggesting uniform incorporation of silver nanowires in the graphene framework, which can not only guide the homogeneous deposition of Li metal, but also form Li_x_Ag, reducing the Li nucleation overpotential [50].

**Figure 2:**
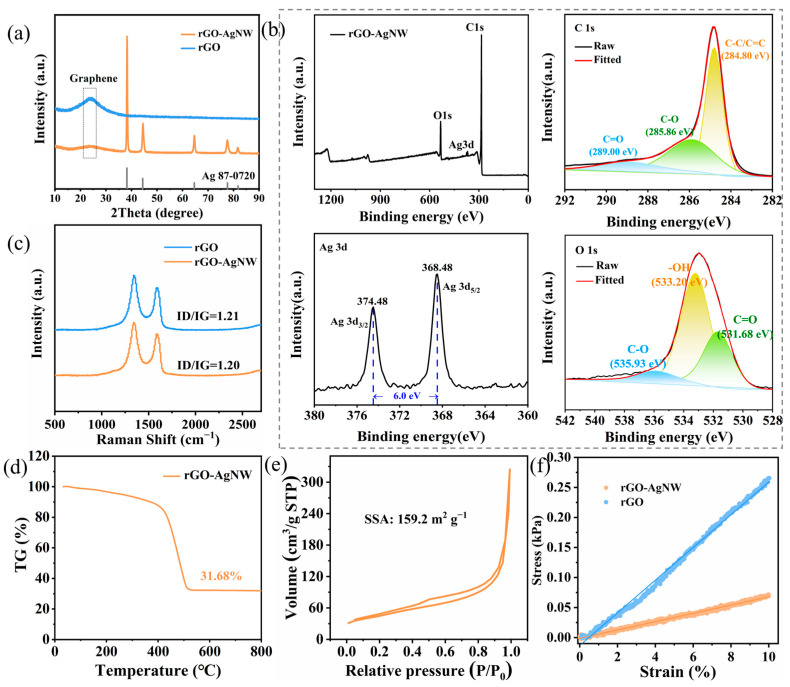
(**a**) XRD patterns of rGO and rGO-AgNW aerogels. (**b**) XPS characterization of rGO-AgNW aerogel. (**c**) Raman spectra of rGO and rGO-AgNW aerogels. (**d**) TG test of rGO-20AgNW in an air atmosphere. (**e**) N_2_ sorption isotherm of rGO-20AgNW aerogel. (**f**) Elastic part of the stress–strain curves of rGO and rGO-AgNW aerogels.

The composition of rGO and rGO-AgNW aerogels were evaluated by X-ray diffraction (XRD) analysis. As shown in Figure 2a, there is a broad peak at 24°, indicating the successful reduction of GO and recovery of graphene layers. The rGO-AgNW aerogel exhibits strong diffraction peaks at 38.2°, 44.4°, 64.6°, 77.6°, and 81.8°, which correspond with different lattice planes of Ag (PDF#87-0720). Furthermore, the peak height of graphene in rGO-AgNW is lower than rGO, which may be due to the weaker diffraction peak intensity of graphene when compared to metal Ag [51]. X-ray photoelectron spectroscopy (XPS) was conducted to characterize the surface chemistry in the rGO-AgNW aerogel. Peak fitting was performed on C1s and O1s curves, respectively. There are three fitting peaks in both graphs, located at 284.80, 285.86, and 289.00 eV in C1s and 533.20, 531.68, and 535.93 eV in O1s. More detailed information is shown in Table S1 and S2. This shows that graphene oxide is partially reduced, and a certain amount of oxygen-containing functional groups remain on the surface, mainly hydroxyl, carboxyl, and lactone groups [52]. The two peaks present in Ag3d are located at 374.48 and 368.48 eV, respectively, with a difference of 6 eV in between, indicating that Ag is not bonded with other elements [53], which is corroborated by the XRD result.

In the Raman spectra of the rGO and rGO-AgNW aerogels (Figure 2c), there are two peaks located near 1350 cm^−1^ (D) and 1580 cm^−1^ (G) [54]. After peak fitting, the calculated ID/IG values are 1.21 and 1.20, respectively, indicating that the silver nanowires have almost no effect on the orderliness of reduced graphene oxide. The result of conducting thermal gravimetric testing on rGO-20AgNW in an air atmosphere is shown in Figure 2d. From room temperature to 400 °C, the weight slowly decreased because of the residual water evaporation and the thermal reduction of oxygen-containing functional groups. The reaction between graphene and oxygen caused a sharp drop in weight in the range of 400–500 °C. The remaining weight (silver) is 31.68% after 800 °C. The BET result is shown as a N_2_ adsorption–desorption isotherm in Figure 2e. The specific surface area of the rGO-AgNW aerogel is calculated to be 159.2 m^2^ g^−1^. The enlarged specific surface area of the rGO-AgNW aerogel can effectively mitigate the localized current density of the electrode, thereby inhibiting dendrite formation [55].

In order to compare the elasticity of the two aerogel materials, compression tests were performed, as shown in Figure 2f. By fitting the curves of elastic deformation, the Young’s modulus of rGO and rGO-20AgNW is 2.72 and 0.69 kPa, respectively. The smaller Young’s modulus indicates that the rGO-20AgNW aerogel has greater elasticity, which can adapt to the volume changes during lithium deposition/stripping processes and reduce internal stress, improving the structural stability of lithium metal anodes. Therefore, the decoration of the 3D lithiophilic rGO-AgNW aerogel current collectors is a productive strategy for improving the mechanical properties of the electrodes and inhibiting dendritic growth.

**Figure 3:**
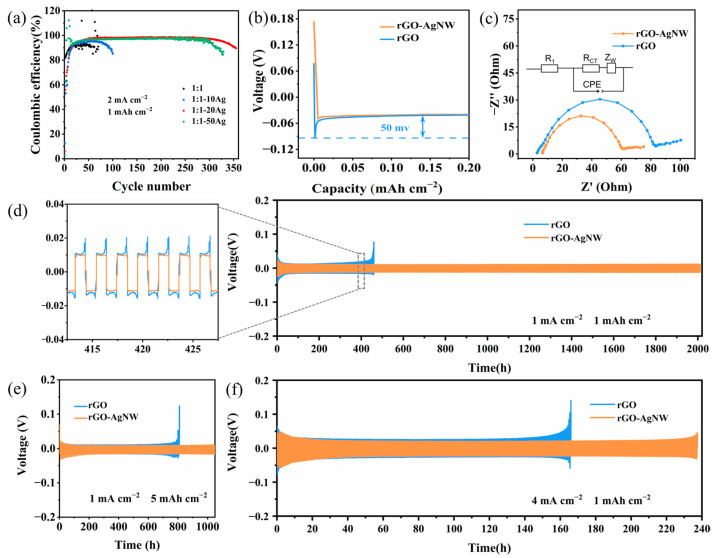
(**a**) Coulombic efficiency at the current density of 2 mA cm^−2^ and the capacity of 1 mAh cm^−2^. (**b**) Corresponding discharge curves in the first cycles of the two electrodes at 2 mA cm^−2^ during CE tests. (**c**) EIS curves of the two symmetric cells. Galvanostatic cycling of symmetric Li/rGO–Li and Li/rGO-AgNW–Li cells at (**d**) 1 mA cm^−2^, 1 mAh cm^−2^, (**e**) 1 mA cm^−2^, 5 mAh cm^−2^, and (**f**) 4 mA cm^−2^, 1 mAh cm^−2^.

To reveal the effect of the silver nanowires, we prepared reduced graphene oxide aerogels with different amounts of silver nanowires and carried out Coulombic efficiency tests at the current density of 2 mA cm^−2^ and the capacity of 1 mAh cm^−2^. As shown in Figure 3a, both rGO and rGO-10AgNW exhibit poor cycling stability due to their poor lithium affinity. The CE of rGO-20AgNW reaches 98.5% and remains stable for over 300 cycles. rGO-50AgNW also possesses good cyclic stability, but its CE is slightly lower, about 97%. As the amount of silver nanowires increases, the rGO-AgNW aerogel provides more lithiophilic sites and induces uniform deposition of lithium, while excessive silver depletes active lithium and reduces the Coulomb efficiency of electrodes. Therefore, 20% silver nanowires was regarded as the ideal additive amount in the rGO aerogel. In Table S3, the preparation method, specific surface area, and performance of rGO-AgNW compared with the published literature is summarized. Moreover, the discharge curves of rGO and rGO-AgNW during Li plating were obtained to explore the nucleation process. As shown in Figure 3b, rGO exhibited a 50 mV Li plating overpotential at the current density of 2 mA cm^−2^. In contrast, the discharge curve of rGO-AgNW is relatively smooth, indicating a small overpotential of 8 mV. Hence, the Ag nanowire-decorated rGO aerogel provided a near-zero Li nucleation barrier. Figure 3c shows the EIS of the symmetric cells before cycling. According to the fitting results, the charge-transfer resistance (R_CT_) of the rGO-AgNW/Li electrode is 53.8 Ω, smaller than that of the rGO/Li electrode (82.3 Ω). This result demonstrates that the rGO aerogel with silver nanowires reduces impedance, which is beneficial for Li diffusion and deposition kinetics.

We conducted long-term galvanostatic cycling of symmetric cells to evaluate the stability of Li plating/stripping. As shown in Figure 3d–f, rGO/Li and rGO-AgNW/Li electrodes both reveal the gradual decrease in polarization under different test conditions. The stable SEI film has not been formed on the surface of the electrode during the initial cycles due to the large specific surface area of the rGO aerogel. As the cycle continues, the electrolytes wet the entire electrode material, a stable SEI film is gradually formed, and the polarization decreases. As seen in Figure 3d, at 1 mA cm^−2^ and 1 mAh cm^−2^, the rGO-AgNW/Li electrode can cycle over 2000 h, demonstrating its extraordinary long-term cycling performance. By contrast, the deterioration of the rGO/Li electrode emerges with the increase in voltage profiles merely after 210 cycles, which can possibly be ascribed to the incessant depletion of electrolytes induced by the irreversible dendritic Li deposition. In the magnification curve of 410–430 h, it can be seen that rGO-AgNW/Li has a stable and low voltage hysteresis of about 10 mV in; however, the polarization of rGO/Li is close to 20 mV. It is worth noting that as the current density increases up to 4 mA cm^−2^, rGO-AgNW/Li and rGO/Li electrodes exhibit stable cycling for about 235 and 160 h, respectively (Figure 3f). Similarly, the rGO-AgNW/Li electrode still presents smaller polarization and better cycle stability than the rGO/Li electrode at the current density of 1 mA cm^−2^ with a higher capacity of 5 mAh cm^−2^. Silver has a very high lithiophilicity, and the nucleation overpotential of lithium metal on silver is approximately zero, so it can provide more lithium deposition sites [38]. At the same time, silver has good electrical conductivity and can accelerate electron transport, thereby enhancing the conductivity of rGO-AgNW. The results indicate that increased lithiophilic active sites and enhanced conductivity of 3D current collectors contribute to extending cycling life.

**Figure 4:**
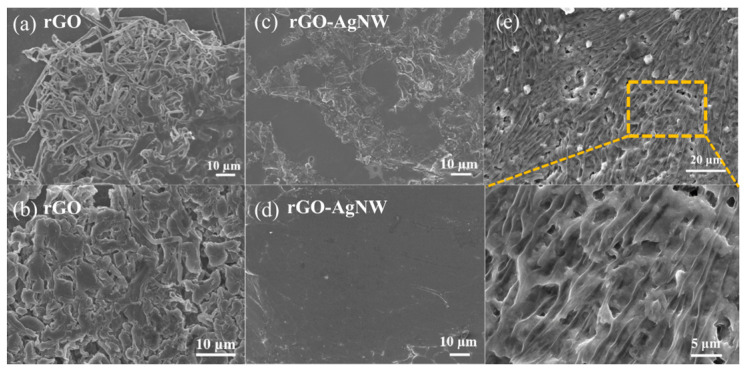
Top-view SEM images of Li plating on rGO and rGO-AgNW electrodes with the capacity of (**a**,**c**) 3 mAh cm^−2^ and (**b**,**d**) 7 mAh cm^−2^ at a current density of 0.5 mA cm^−2^. (**e**) SEM images of rGO-AgNW/Li in a symmetric cell after 100 cycles complete lithium removal.

In order to understand the effect of silver nanowire-decorated 3D rGO aerogels on inducing lithium deposition and inhibiting dendrite growth, different capacities of lithium were deposited on the rGO and rGO-AgNW skeletons at a current density of 0.5 mA cm^−2^. Obvious lithium dendrites are clearly observed on the surface of rGO when depositing 3 mAh cm^−2^ of lithium (Figure 4a). This is because the surface of rGO is lithiophobic, the overpotential of lithium nucleation on its surface is high, and lithium tends to grow along the top of the deposited lithium to form dendrites. When the deposition of lithium increases to 7 mAh cm^−2^, the surface of rGO is covered by loosely packed lithium dendrites (Figure 4b). Contrary to this, the Li deposition on the rGO-AgNW electrode shows a smooth and compact morphology (Figure 4c,d). Lithiophilic AgNW fixed on the graphene sheets is evenly dispersed in the skeleton, providing sufficient nucleation sites to induce lithium deposition in the pores. In addition, silver nanowires with good lithiophilicity can uniform lithium ion flux, thus inducing uniform lithium deposition [38]. In order to observe the morphology of the skeleton after cycling, the rGO-AgNW/Li electrode was completely delithiated after 200 cycles. As shown in Figure 4e, the surface presents a textured appearance, which is formed by silver nanowires, suggesting a stable structure of rGO-AgNW.

**Figure 5:**
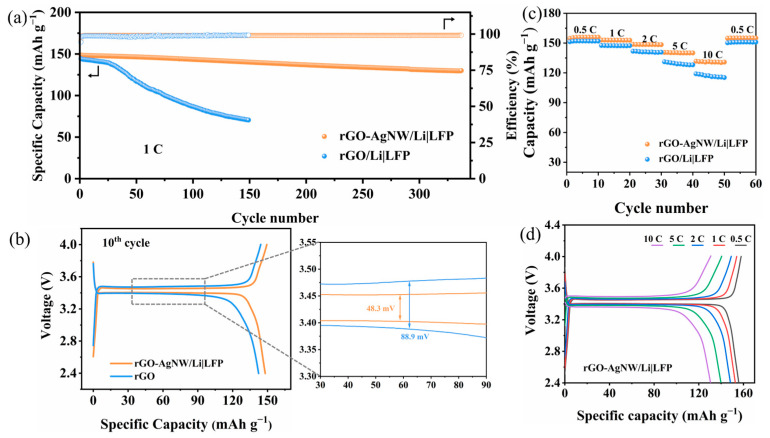
(**a**) Cycling performance of rGO-AgNW/Li|LFP and rGO/Li|LFP cells at 1 C. (**b**) Charge and discharge curves and the partial enlarged view of the full cells in the 10th cycle. (**c**) Rate performance of rGO-AgNW/Li|LFP and rGO/Li|LFP cells and (**d**) corresponding voltage profiles of rGO-AgNW/Li|LFP cell.

Full cells for rGO-AgNW/Li and rGO/Li electrodes coupled with LiFePO_4_ (LFP) as cathodes were assembled and tested to illustrate the performance in practical applications. Figure 5a shows the long-term cycling performance of the full cells at 1 C. The rGO/Li|LFP cell exhibits a slow capacity decrease for the first 30 cycles, after which it shows a sharp capacity decline due to the serious loss of active Li-ion. On the contrary, the rGO-AgNW/Li|LFP operates steadily over 350 cycles and realizes a great capacity retention of 87.22%. Charge/discharge platforms and voltage hysteresis in the 10th cycle and the enlarged view are shown in Figure 5b. The rGO-AgNW/Li|LFP full cell possesses a voltage hysteresis of 48.3 mV, smaller than rGO/Li|LFP (88.9 mV). The accumulation of “dead” Li during cycling leads to larger polarization of the rGO/Li|LFP full cell. Notably, the rGO-AgNW/Li|LFP cell also shows a better rate performance as presented in Figure 5c,d and a high capacity of 130 mAh g^−1^ can be obtained at a high rate of 10 C.

The lightweight 3D lithiophilic graphene aerogels as current collectors represent a significant advancement in the development of next-generation high-energy-density lithium metal batteries. These aerogels possess exceptional properties such as high porosity, large surface area, and excellent electrical conductivity, which are crucial for enhancing the performance and stability of lithium metal anodes. Furthermore, the integration of graphene aerogels with Ag nanowires as current collectors opens up new possibilities for the development of lightweight and flexible battery architectures, enabling the realization of compact and portable energy storage devices for various consumer electronics, electric vehicles, and renewable energy systems. Therefore, the exploration of lightweight aerogels as current collectors in lithium metal batteries expands strategies for advancements in battery technology.

## 4. Conclusions

In summary, a graphene aerogel decorated with homogeneous silver nanowires was fabricated via a facile chemical reduction method. The rGO-AgNW aerogel has the following advantages: (1) With high specific surface area of 3D current collectors, it can reduce local current density of electrodes. (2) The interconnected three-dimensional porous structure provides space for lithium deposition and adapts to the volume change in the lithium plating/stripping process. (3) Strong lithiophilicity reduces the nucleation overpotential of lithium. (4) High elasticity and a small Young’s modulus help to adapt to the stress caused by volume changes. In addition, the rGO-AgNW aerogel also possesses lightweight characteristics, which can effectively improve the anode energy density when acting as a host for lithium deposition. Owing to these prominent advantages, the rGO-AgNW displays a high Coulombic efficiency of 98.5% over 300 cycles at 2 mA cm^−2^ and cycles for over 2000 h with a stable and low voltage hysteresis of 10 mV at 1 mA cm^−2^. Full cells also exhibit good electrochemical performance, with a capacity retention of 87.22% after 350 cycles. This work can offer valuable insights into the design of Li metal anodes for the next-generation high-energy-density lithium metal batteries. 

## Data Availability

The original contributions presented in the study are included in the article/Supplementary Material, further inquiries can be directed to the corresponding authors.

## Supplementary Materials

The following supporting information can be downloaded at: https://www.mdpi.com/article/10.3390/ma17071693/s1, Figure S1: Photos of (a) the front and (b) side of the sample; Figure S2: The weight of different materials, (a) Cu foil, (b) rGO aerogels with 20% AgNWs and (c) pure rGO aerogels; Table S1: Peak fitting result of C1s; Table S2: Peak fitting result of O1s; Table S3: The comparison of different 3D current collectors.
