# Supplementary material for: Lightweight 3D Lithiophilic Graphene Aerogel Current Collectors for Lithium Metal Anodes
Source: Materials (Basel). 2024 Apr 7;17(7):1693. doi: 10.3390/ma17071693 (PMC11012320; doi:10.3390/ma17071693)
Supplement: Supplementary file 1 [file materials-17-01693-s001.zip › materials-2936853-supplementary.pdf]

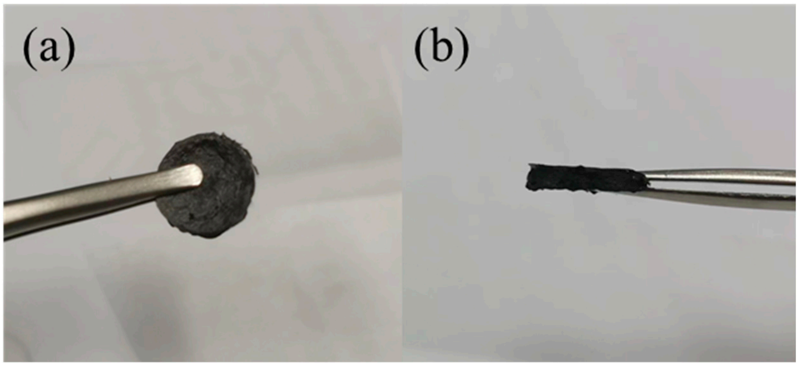

Figure S1. Photos of (a) the front and (b) side of the sample.

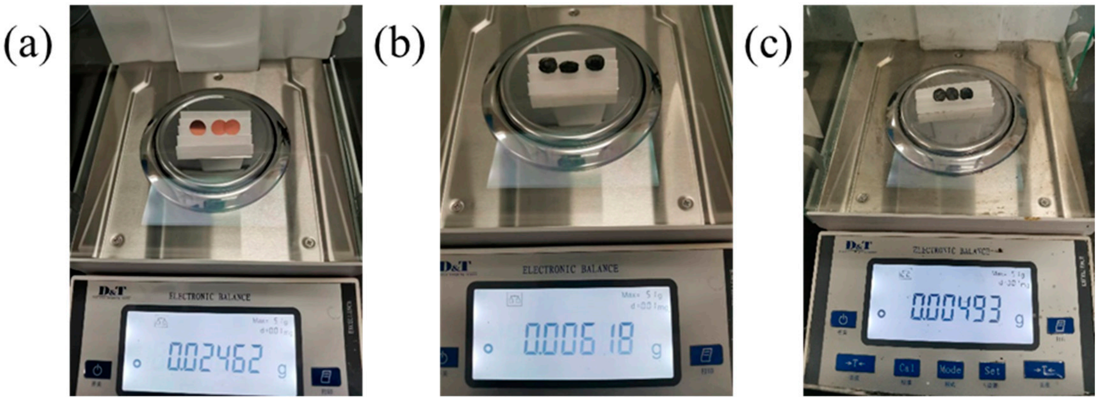

Figure S2. The weight of different materials, (a) Cu foil, (b) rGO aerogels with 20% AgNWs and (c) pure rGO aerogels.

The copper foil was cut into a diameter size close to that of reduced graphene oxide aerogel. As shown in Figure S2, the weight charts of three materials show that pure rGO aerogel is the lightest, about 20% of copper foil, followed by rGO aerogel with 20% AgNWs, about 25% of copper foil. This demonstrates the excellent lightweight nature of the reduced graphene oxide aerogel materials, which contributes to improve the energy density of lithium metal anodes.

Table S1. Peak fitting result of C1s.

Name	Binding energy (eV)	FWHM (eV)	Percentage (%)
C-C/C=C	284.80	1.01	51.81
C-O	285.86	2.84	36.79
C=O	289.00	3.33	11.40

Table S2. Peak fitting result of O1s.

Name	Binding energy (eV)	FWHM (eV)	Percentage (%)
-OH	533.20	2.38	62.50
O=C	531.68	2.37	31.25
-O-C	535.93	2.85	6.25

Table S3. The comparison of different 3D current collectors.

Current collector	Preparation method	SSA (m^2 g^{-1})	Half cell performance (Cycle number, CE, Current density)	Refs
Ni_3N@NS	Powder metallury	3.332	350, 98.8%, 2	[1]
Crumpled MXene	Blade-coating	107.28	160, 97%, 1	[2]

CNT scaffold	Vacuum filtration	221	150, 90%, 1	[3]
AgNW aerogel	Modified polyol reduction	6.7	100, 98%, 0.5	[4]
RGO-AgNW aerogel	Chemical reduction	159.2	300, 98.5%, 2	This work
